# Socio-demographic and psychosocial characteristics of male and female perpetrators in intimate partner homicide: A case-control study from Region Västra Götaland, Sweden

**DOI:** 10.1371/journal.pone.0256064

**Published:** 2021-08-31

**Authors:** Linnea Carlsson, Henrik Lysell, Viveka Enander, Karin Örmon, Solveig Lövestad, Gunilla Krantz

**Affiliations:** 1 The Västra Götaland Region Competence Centre on Intimate Partner Violence, Gothenburg, Sweden; 2 Occupational and Environmental Medicine, The Sahlgrenska Academy, Gothenburg, Sweden; 3 Swedish National Board of Health and Welfare, Stockholm, Sweden; 4 Department of Social Work, University of Gothenburg, Gothenburg, Sweden; 5 Department of Care Science, Faculty of Health and Society, Malmö University, Malmö, Sweden; 6 School of Public Health and Community Medicine, Institute of Medicine, University of Gothenburg, Gothenburg, Sweden; Universidad Pública de Navarra, SPAIN

## Abstract

Risk factor studies on male-perpetrated intimate partner homicide (IPH) are often compared with studies on intimate partner violence (IPV) or non-partner homicide perpetrators. This not only excludes female perpetrators, but also fails to take socio-demographic and psychosocial differences between perpetrators and the general population into consideration. The aim of this study was to examine male- and female-perpetrated IPH cases, and to compare socio-demographic factors in IPH perpetrators and in matched controls from the general population. Data were retrieved from preliminary inquiries, court records and national registers for 48 men and 10 women, who were perpetrators of IPH committed in 2000–2016 and residing in Region Västra Götaland, Sweden. The control group consisted of 480 men and 100 women matched for age, sex and residence parish. Logistic regression, yielding odds ratios (OR) with 95% confidence intervals (CI), was performed for male perpetrators and male controls to investigate associations for selected socio-demographic and psychosocial characteristics. This was not performed for females due to the small sample size. Female perpetrators were convicted of murder to a lesser extent than male perpetrators. No woman was sentenced to life imprisonment while five men were. Jealousy and separation were the most common motivational factors for male perpetration while the predominant factor for female perpetrators was subjection to IPV. Statistically significant differences were found between male perpetrators and male controls in unemployment rate (n = 47.9%/20.6%; OR 4.4; 95% CI 2.2–8.6), receiving benefits (n = 20.8%/4.8%; OR 5.2; 95% CI 2.3–11.7) and annual disposable income (n = 43.8%/23.3% low income; OR 5.2; 95% CI 1.9–14.2) one year prior to the crime. Female IPH perpetrators were less educated than female controls (≤ 9-year education 30%/12%) and were more often unemployed (70%/23%) one year before the crime. Male and female IPH perpetrators were socio-economically disadvantaged, compared with controls from the general population.

## Introduction

Globally, one in seven homicides (13.5%) is committed by an intimate partner [1]. Of all homicides, the proportion of women killed by a current or former intimate partner is six times higher (38.6%) than the corresponding proportion of men (6.3%) [1]. In Sweden, intimate partner homicide (IPH) accounts for 24% of all homicides [2] and roughly 80% of victims are women killed by their male current or former partners [3,4]. Previous research from Sweden has shown that 15 to 17 women have been killed each year during the past decade by a current or former male partner, whereas 3–5 men have been killed by a female current or former partner [3,4].

Male IPH perpetration is commonly preceded by intimate partner violence (IPV) directed at a current or former partner [1]. Identified major risk factors are jealousy and possessiveness, often as part of a pattern of coercive control, and prior abuse [3,5,6]. Dobash & Dobash also showed that increased socio-demographic disparities, due to women’s higher educational level, income, employment status and/or rank, may challenge men’s superior position in the family, which may lead to male-perpetrated IPH [7]. This is connected to what has been described as the Nordic paradox, i.e., that in a country with a relatively high level of gender equality on the structural level, there are still difficulties on the individual level relating to this situation [8]. Furthermore, mental problems or altered life circumstances, such as divorce or (threat of) separation, often trigger severely violent acts, resulting in IPH. These homicides are often the ultimate outcome of a failed response to IPV from society, the healthcare sector or the criminal justice system [1].

Regarding female perpetration, women generally kill in self-defence or in retaliation for IPV, after having been systematically victimised over longer periods of time, as confirmed by an extensive body of research [9–11]. Occasionally, however, female perpetrators commit IPH driven by apparently unprovoked aggression, and in some few cases women systematically commit IPV against a male partner over a longer period of time [9].

Researchers examining IPH perpetrators and related socio-demographic factors mainly compare their findings with those from studies on perpetrators of IPV or non-partner homicide. Lower educational level, economic disadvantage and unemployment are more commonly observed among male IPH perpetrators, compared with male IPV perpetrators [4,5,7,10,12,13]. Comparing perpetrators of IPH with perpetrators of non-partner homicide reveals that IPH perpetrators are more educated and to a higher extent in paid employment, and thus more “conventional” and comparable to the general population [4,14]. This is a useful approach in attempting to discern what distinguishes IPH perpetrators from other violence perpetrators, but it fails to assess risk factors related to the IPH perpetrators in comparison with the general population, which might be useful for policy initiatives and improved prevention.

This study is part of the IPH-STOP study, a comprehensive research endeavour on all cases of IPH occurring in the western part of Sweden during 2000–2016. IPH-STOP comprises studies comparing IPH perpetrators and control groups representing the general population, regarding different parameters such as healthcare service attendance, medication intake and criminal records. Other IPH-STOP studies include thematic analyses and qualitative interviews with close relatives of victims and perpetrators. The overarching aim of this project is to identify risk factors for IPH, which might possibly contribute to prevention.

The aims of this particular case-control study were:

1. To investigate and present the crime-related circumstances in male- and female-perpetrated IPH cases, based on data from preliminary inquiries and court records
2. To compare socio-demographic factors in male-perpetrated IPH cases with those in male controls drawn from a general population sample
3. To compare socio-demographic factors in female-perpetrated IPH cases with those in female controls drawn from a general population sample

## Materials and methods

### Definition of IPH

IPH refers to the intentional use of violence that leads to death, including manslaughter, involuntary manslaughter and murder. According to the National Council for Crime Prevention (BRÅ), IPH includes deeds committed by a current or former spouse, partner or boy-/girlfriend [15].

### Study population

The study population consisted of 48 male perpetrators and 10 female perpetrators, and included one perpetrator of male-to male IPH. Among the male perpetrators, one individual killed two partners on two different occasions, thus yielding a total of 49 male-perpetrated cases. Among the 48 male perpetrators, eight committed suicide immediately or soon after the deed, referred to as Intimate Partner Homicide-Suicide (IPHS). All perpetrators were aged over 18, an eligibility criterion, and included in this study irrespective of gender, marital status or sexual orientation.

Each perpetrator was randomly matched with ten controls from the general population, based on age, sex and residence parish, resulting in a total sample (IPH subsample plus control sub sample) of 638 individuals. Statistics Sweden (SCB) provided this data.

### Data collection

Data for this study were retrieved from two main source types: preliminary inquiries and court records from the regional police authority and the national registers from SCB and the Swedish National Board of Health and Welfare (NBHW). The NBHW is a government agency under the Swedish Ministry of Health and Social Affairs, acting within the fields of social services, healthcare and medical services, patient safety and epidemiology.

The perpetrators, victims and their children were identified from the court records and the preliminary inquiries, and some personal data were retrieved. The court records contain detailed descriptions of events preceding, during and following the IPH, motivational factors as described by the perpetrators and witnesses, if any, as well as verdicts and sentences. The preliminary inquiries were used for information on the identified IPHS cases. The national registers mentioned above store data on all individuals who are officially registered residents of Sweden aged over 16 on December 31 each year.

Based on the personal data found in the court records, SCB provided socio-demographic and psychosocial information on all study subjects, i.e., perpetrators and controls, via the 10-digit personal identification number (PIN) provided to all residents at birth or upon official registration of residence in Sweden. SCB replaced all the subjects’ PINs with serial numbers, with encoding unavailable to the researchers. This enabled linkage of the different registers, and guaranteed anonymity of the subjects. Data on receiving financial social benefits were obtained from the NBHW. Furthermore, data, including the socio-demographic characteristics of perpetrators and controls, were retrieved from SCB’s Longitudinal Database for Integration Studies (STATIV) [16] and the Longitudinal Integration Database for Health Insurance and Labour Market Studies (LISA) [17].

### Variables

A template was developed for systematically collecting information from the court records or, in the IPHS cases, from the preliminary inquiries. All researchers read through all court records and preliminary inquiries and compiled the information in a database. In this way, data which is unretrievable from official national registers could be accessed and presented (Table 1). For statistical significance testing of differences between male and female perpetrators, variables were dichotomised. How dichotomisation was done is indicated in Table 1.

**Table 1:** Descriptive data related to IPH cases, numbers (n) and percentages (%), with Fisher’s exact test for male-perpetrated and female-perpetrated cases with dichotomised variables. N = 48 male and 10 female perpetrators, total of 59 cases*.

Variables based on perpetrated cases, including suicide cases	Male homicide cases, n = 49*		Female homicide cases, n = 10		Dichotomised variables	Male homicide cases, n = 49*		Female homicide cases, n = 10		Fischer Exact Test, 2-sided
	n	%	n	%		n	%	n	%	
**Suicide after homicide**	8	16.3	0	00						
**Relationship at homicide**					**Relationship**					
Spouse **	16	32.7	2	20.0	Spouse	29	59.2	4	40.0	.311
Cohabiting partner **	13	26.5	2	20.0	Other	20	40.8	6	60.0	
Non-cohabiting partner	6	12.2	4	40.0						
Divorced/separated	9	18.4	1	10.0						
Other	5	10.2	1	10.0						
**Length of relationship before divorce/separation or homicide**					**Length of relationship**					
< I year	4	8.2	2	20.0	1–5 years	19	47.5	5	50	1.000
1–5 years	15	30.6	3	30.0	> 5 years	21	52.5	5	50	
6–10 years	4	8.2	1	10.0						
>10 years	17	34.7	4	40.0						
No information	9	18.4	0	0.0						
**Ongoing or conceivable divorce/separation**					**Ongoing divorce/separation**					
Yes	20	40.8	3	30.0	Yes	20	40.8	3	30.0	.725
No	29	59.2	7	70.0	No	29	59.2	7	70.0	
**Homicide location**					**Homicide location**					
At home	38	77.6	7	70.0	At home	38	77.6	7	70.0	.688
Workplace	2	4.1	0	0.0	Other places	11	22.4	3	30.0	
Outdoors	7	14.3	1	10.0						
Other	2	4.1	2	20.0						
**Children present at time of homicide**					**Children present**					
Victim of violence	1	2.0	0	0.0	Present	13	35.1	2	28.6	1.000
Witnessed homicide	6	12.2	1	10.0	Not present	24	64.9	5	71.4	
At home but did not witness homicide	6	12.2	1	10.0						
Not present	24	49.0	5	50.0						
No information	12	24.6	3	30.0						
**Variables based on perpetrated cases, excluding suicide cases**	**Male homicide cases, N = 41**		**Female homicide cases, N = 10**		**Dichotomised variables**	**Male homicide cases, n = 48**		**Female homicide cases, N = 10**		**Fischer Exact test, 2-sided**
	n	%	n	%		n	%	n	%	
**Verdict in district court**					**Verdict in district court**					
Murder	35	85.4	5	50.0	Murder	35	85.4	5	50.0	.027
Manslaughter	1	2.4	3	30.0	All other	6	14.6	5	50.0	
Involuntary manslaughter	3	7.3	1	10.0						
Not guilty	2	4.9	1	10.0						
**Verdict in higher court**					**Verdict in higher court**					
Murder	36	87.8	5	50.0	Murder	36	87.8	5	50.0	.017
Manslaughter	2	4.9	2	20.0	All other	5	12.2	5	50.0	
Involuntary manslaughter	3	7.3	2	20.0						
Not guilty	0	0.0	1	10.0						
**Sentence in higher court**					**Sentence in higher court**					
Life imprisonment	6	14.6	0	0.0	Life imprisonment	6	14.6	0	0.0	.331
Fixed-term imprisonment	22	53.7	9	90.0	Fixed-term imprisonment	22	53.7	9	90.0	.067
Forensic psychiatric care	11	26.8	0	0.0	Forensic psychiatric care	11	26.8	0	0.0	.094
Deportation ***	4	9.8	0	0.0	Deportation ***	4	9.8	0	0.0	.573
**Mode of killing†**					**Mode of killing†**					
Knife	19		6		Knife/Other	19	39.6	6	60.0	.499
*Number of stabs*:					-					
*1–2*	*5*	*-*	3	-	-					
*3–5*	*5*	*-*	0	-	-					
*>10*	*5*	*-*	2	-	-					
*No information on record*	*4*	*-*	1	-	-					
Firearm	8		0	-	Firearm/Other	8	16.7	0	0.0	.329
Strangulation	7		1	-	Strangulation/Other	7	14.6	1	10.0	1.000
Blunt violence or other	11		3	-	Blunt violence/Other	11	22.9	3	30.0	1.000
**Motivational factors †**					**Motivational factors †**					
Jealousy	15	-	2	-	Jealousy/Other	15	36.6	2	20.0	.463
Separation	16	-	3	-	Separation/Other	16	39.0	3	30.0	.725
Subjected to IPV	1	-	5	-	Subjected to IPV/Other	1	2.4	4	40.0	.004
Financial conflict	3	-	3	-	Financial conflict/Other	3	7.3	3	30.0	.081
Custody dispute	1	-	0	-	Custody dispute/Other	1	2.4	0	0.0	1.000
Other	17	-	0	-						

* There were 49 male-perpetrated cases if IPHS were included and 41 male-perpetrated cases if IPHS were excluded, respectively, as one individual had killed two ex- partners.

** One married individual was not cohabiting with the partner and one cohabiting couple had separated at the time of the homicide.

*** Two individuals were sentenced to life imprisonment + deportation and two individuals were sentenced to fixed-term imprisonment + deportation.

**†** More than one mode of killing and motivational factors were reported in the court records; numbers (n) and percentage (%) do thus not correspond with the total n.

Socio-demographic and psychosocial factors analysed for male and female perpetrators and controls comprise age, sex, marital status, income, employment status, receiving social benefits, highest educational level and housing standard. Disposable annual income was divided into three groups: high (>253.250 SEK), medium (117.775–253.250 SEK) and low (<117.775 SEK). The social benefits variable includes all individuals who received financial benefits for at least one month and up to one year.

Age and parents’ highest level of education were extracted for the same year as the occurrence of the crime. All other data were extracted for a time point one year preceding the crime for both perpetrators and controls. Employment status is recorded for the month of November each year and was extracted for index-1 for the purposes of this study. The income and social benefits factors also included data from a time point three years preceding the crime (index-3), which was used to strengthen the validity of the results.

All variables, both continuous and nominal, were computed and dichotomised with respect to the small sample size. Rules for dichotomisation followed the recommendations and coding principles provided by SCB [16,17] and NBHW [18].

### Statistical procedures

Descriptive data on male and female IPH cases are presented with numbers (n) and percentages (%), p-values for differences are presented for dichotomised variables, calculated with Fisher’s exact two-sided test (Table 1).

Differences in the distribution of socio-demographic and psychosocial variables between perpetrators and controls were calculated with Fisher’s exact two-sided test, separately for males and females (Tables 2 and 4). The significance level was set at five percent, and a p-value below 0.05 indicated a statistically significant difference between the perpetrators and controls, with males and females analysed separately. Variables exhibiting a statistically significant difference between male perpetrators and male controls underwent further analyses. Bivariable associations were analysed by use of logistic regression, yielding odds ratios (OR) with 95% confidence intervals (CI), to investigate associations between each of the independent variables and the observed outcome of interest, i.e., differences in socio-demographic characteristics between perpetrators and controls (Table 3). Due to the small female-perpetrator sample size (10 individuals), further analyses were not performed for this group. According to Green’s [19] recommendation, the sample size of 48 male perpetrators is too small to perform multivariable analysis. Another reason for not performing multivariable analysis was that the statistically significant socio-demographic variables in the bivariable analysis were highly correlated, and an adjusted regression analysis would not have contributed any new information.

**Table 2:** Socio-demographic characteristics of male perpetrators, male controls and the total population. NN = 528, 48 perpetrators and 480 controls.

Factors	Male perpetrators n = 48	Male controls n = 480	Total	Fisher’s exact 2-sided test
	n (%)	n (%)	n (%)	
Age*				
20–30	6 (12.5)	60 (12.5)	66 (12.5)	
31–40	13 (27.1)	130 (27.1)	143 (27.1)	1
41–50	10 (20.8)	100 (20.8)	110 (20.8)	
51–60	11 (22.9)	110 (22.9)	121 (22.9)	
≥61	8 (16.7)	80 (16.7)	88 (16.7)	
Marital status**				
Married/registered partner	17 (35.4)	216 (45.0)	233 (44.1)	
Unmarried	17 (35.4)	196 (40.8)	213 (40.3)	0.108
Divorced/widower	12 (25)	67 (14)	79 (15)	
Missing	2 (4.2)	1 (0.2)	3 (0.6)	
Education**				
Pre-secondary, ≤9 years	9 (18.8)	93 (19.4)	102 (19.3)	
Secondary, 9–12 years	28 (58.3)	223 (46.5)	251 (47.5)	0.079
Post-secondary, > 12 years	6 (12.5)	128 (26.7)	134 (25.4)	
Missing	5 (10.4)	36 (7.5)	41 (7.8)	
Housing standard**				
House	17 (35.4)	210 (43.8)	227 (43)	
Owned apartment	7 (14.6)	56 (11.7)	63 (11.9)	0.408
Rented apartment	15 (31.3)	122 (25.4)	137 (25.9)	
Missing	9 (18.8)	92 (19.2)	101 (19.1)	
Disposable annual income**				
Low	21 (43.8)	112 (23.3)	133 (25.2)	
Medium	20 (41.7)	228 (47.5)	248 (47)	0.001
High	5 (10.4)	139 (29)	144 (27.3)	
Missing	2 (4.2)	1 (0.2)	3 (0.6)	
Receiving benefits**				
Yes	10 (20.8)	23 (4.8)	33 (6.3)	<0.001
No	38 (79.2)	457 (95.2)	495 (93.8)	
Employment status**				
Employed	16 (33.3)	300 (62.5)	316 (59.8)	
Unemployed	23 (47.9)	99 (20.6)	122 (23.1)	<0.001
Missing	9 (18.8)	81 (16.9)	90 (17)	
Education, mother***				
Pre-secondary, ≤9 years	13 (27.1)	144 (30)	157 (29.7)	
Secondary, 9–12 years	12 (25)	137 (28.5)	149 (28.2)	0.224
Post-secondary, > 12 years	1 (2.1)	56 (11.7)	57 (10.8)	
Missing	22 (45.8)	143 (29.3)	165 (31.3)	
Education, father***				
Pre-secondary, ≤9 years	9 (18.8)	118 (24.6)	127 (24.1)	
Secondary, 9–12 years	11 (22.9)	125 (26)	136 (25.8)	0.866
Post-secondary, > 12 years	2 (4.2)	38 (7.9)	40 (7.6)	
Missing	26 (54.2)	199 (33)	225 (42.6)	

*Age in index year.

** Figure valid one year before the crime occurred.

*** Highest registered level of education.

**Table 3:** Bivariable associations between selected socio-demographic factors for male perpetrators and male controls, crude odds ratios (OR) with 95% confidence intervals (CI). N = 528; 48 perpetrators and 480 controls.

	Index-1			Index– 3		
Factors	OR	95% CI	p-value	OR	95% CI	p-value
**Disposable annual income**						
High	1					
Medium	2.4	0.89–6.6	0.081	5.3	1.5–18.1	0.007
Low	5.2	1.9–14.2	0.001	6.6	1.9–23.4	0.003
**Receiving benefits** No	1					
Yes	5.2	2.3–11.7	<0.001	5.8	2.7-12-5	<0.001
**Employment status**						
Employed	1					
Unemployed	4.4	2.2–8.6	<0.001			

Missing data was excluded from all analyses. Data management and analysis were performed with IBM SPSS Statistics 25 for Windows.

### Ethical considerations

The perpetrators’ identities were retrieved from official court records and preliminary inquiries that are accessible to researchers upon request to the police authority, if ethical approval is provided. Ethical approval was provided by the Regional Ethical Review Board in Gothenburg (approval number Dnr: 434–16). Access to the sociodemographic data was applied for from population-based registers, managed by SCB and the NBHW. SCB anonymised all data so that no subject could be identified. The registers used for this study are open to researchers upon request if ethical approval has been provided and the request is deemed appropriate by the authority maintaining the register.

## Results

As mentioned above, there were 49 male-perpetrated IPH cases but 48 male perpetrators, since one male perpetrator had killed two women. Furthermore, there were 10 female-perpetrated IPH cases and 10 female perpetrators; the ratio of males to females was thus 82.8%/17.2%. Table 1 depicts the number of cases rather than perpetrators. Thereafter, results are presented based on the number of IPH perpetrators. One of the male-perpetrated IPHs was committed within a same-sex relationship. The mean age was 44.6 years (range 19–88) for male perpetrators and 43.1 years (range 21–59) for female perpetrators. The mean number of IPH events per year was 3.4, 2.8 male-perpetrated and 0.6 female-perpetrated, between 2000 and 2016, in a population of 1.7 million (Västra Götaland Region). This is proportionate to the reported national incidence for Sweden, that has 10.2 million inhabitants [2].

The results, related to the three aims described above, are presented below.

### Male- and female-perpetrated cases based on preliminary inquiries and court records

The data presented in Table 1 illustrates differences between male- and female-perpetrated IPH cases, presented as frequencies with p-values for differences, and dichotomised variables were used. This data was retrieved from preliminary inquiries and court records. The focus is exclusively on the IPH event, and based solely on the data available in these documents. It is important to note that the data concerning motivational factors mainly reflects the perpetrators’ views.

There were no cases of female-perpetrated IPHS, while 16.3% of the male perpetrators committed suicide in conjunction with the IPH. The majority (21 cases) of both male and female perpetrators had been in the relationship for more than 10 years, while the relationship was of short duration (<1 year) in six cases (Table 1). The location of the homicides was mainly the home of the couple or of either of the parties (n = 38). The majority of both male- and female-perpetrated IPH were committed with knives.

The majority of the perpetrators (36 males and 5 females) were convicted of murder in the higher court, while nine (five males and four females) were convicted of manslaughter or involuntary manslaughter.

The sentence was life imprisonment in five of the male-perpetrated cases. Twenty-one male perpetrators were given fixed-term sentences, ranging from 2.5 to 18 years, and 11 were sentenced to forensic psychiatric care with no time limit. Four male perpetrators were also sentenced to deportation from Sweden after having served their prison terms.

All female perpetrators were given fixed-term sentences, ranging between 3 and 16 years, except in one case in which the verdict was “not guilty”, as the deed was deemed to have been committed in self-defence. No female perpetrator was thus sentenced to forensic psychiatric care (Table 1).

Information on the stated motivational factors for the IPH was incomplete. However, jealousy and threat of divorce/separation were quite frequently (31 cases) stated in the court records when it came to male perpetrators. Jealousy (n = 2) and (threat of) separation (n = 3) were part of the motivational background in some female-perpetrated cases as well. However, the female perpetrator had not wanted to separate in only one case, while the other two had initiated the divorce/separation. Four female perpetrators stated being subject to IPV as the main motive (Table 1).

Statistically significant differences between male and female perpetrators were found for verdict in district and higher court with males being convicted to murder to a higher extent than women when compared to all other verdicts. Furthermore, female perpetrators were to a higher extent exposed to IPV (motivational factors) before the killing than male perpetrators.

The data presented below emanates from SCB and the NBHW.

### Socio-demographic factors: Male perpetrators compared to male controls

Comparison of disposable annual income, receiving benefits and employment status revealed statistically significant differences between male perpetrators and male controls, while comparisons of the remaining variables did not. Table 2 shows that 43.8% (n = 21) of the male perpetrators had low disposable income, compared to 23.3% of the male controls. Medium disposable income differed slightly between perpetrators and controls. Moreover, only 10.4% of the male perpetrators had a high disposable income, versus 29% of the controls (Table 2).

Regarding employment status, 33% of the male perpetrators were employed one year prior to the crime, in comparison to nearly 63% of the male controls. The proportion of unemployment among the perpetrators was 47.9%, while it was 20.6% among the controls. Furthermore, 20.8% of the male perpetrators were receiving social benefits one year prior to the crime, whereas the corresponding figure for male controls was 4.8% (Table 2).

In the next step, bivariable analyses among the male group for the index-1-year time-point are presented in Table 3. As data on disposable annual income and benefits were also available for the index-3-year time point, Fisher’s exact test was performed for both time points and statistically significant differences (p-values <0.05) were found for both.

There was a statistically significant association between low disposable annual income the year before the crime occurred (OR = 5.2; 95% CI 1.9–14.2) for male IPH perpetrators, compared with the control group. Furthermore, the male perpetrators had 5.2 (95% CI of 2.3–11.7) times higher odds of being social benefits recipients, compared to controls. The perpetrators also had 4.4 (95% CI 2.2–8.6) times higher odds of having been unemployed one year before the crime was committed, compared to the control group.

When the variables income and receiving social benefits three years prior to the crime (index- 3) were added, complementary findings emerged (Table 4). The odds were high of the perpetrators being in the low (OR 6.6; 95% CI 1.9–23.4) or medium (OR 5.3; 95% CI 1.5–18.1) income groups, compared with controls. This was also the case when it came to receiving social benefits (OR 5.8; 95% CI 2.7–12.5).

**Table 4:** Socio-demographic characteristics of female perpetrators, female controls and total population. N = 110; 10 perpetrators and 100 controls.

Factors	Perpetrators n = 10	Controls n = 100	Total n 110	Fisher’s exact 2-sided test
	n (%)	n (%)	n (%)	
Age*				
20–30	1 (10.0)	10 (10.0)	11 (11.0)	
31–40	3 (30.0)	30 (30.0)	33 (30.0)	1
41–50	3 (30.0)	30 (30.0)	33 (30.0)	
51–60	3 (30.0)	30 (30.0)	33 (30.0)	
≥61	0 (0.0)	0 (0.0)	0 (0.0)	
Marital status**				
Married/registered partner	3 (30.0)	49 (49.0)	52 (47.3)	
Unmarried	5 (50.0)	36 (36.0)	41 (37.3)	0.493
Divorced/widow	2 (20.0)	14 (14.0)	16 (14.5)	
Missing	-	1 (1.0)	1 (0.9)	
Education**				
Pre-secondary, ≤9 years	3 (30.0)	12 (12.0)	15 (13.6)	
Secondary, 9–12 years	6 (60.0)	48 (48.0)	54 (49.1)	0.015
Post-secondary,> 12 years	0 (0.0)	38 (38.0)	38 (34.5)	
Missing	1 (10.0)	2 (2.0)	3 (2.7)	
Housing standard**				
House	3 (30.0)	47 (47.0)	50 (45.5)	
Owned apartment	0 (0.0)	15 (15.0)	15 (13.6)	0.124
Rented apartment	7 (70.0)	36 (36.0)	43 (39.1)	
Missing	-	2 (2.0)	2 (1.8)	
Disposable annual income**				
Low	4 (40.0)	22 (22.0)	26 (23.6)	
Medium	6 (60.0)	63 (63.0)	69 (62.7)	0.321
High	0 (0.0)	14 (14.0)	14 (12.7)	
Missing	-	1 (1.0)	1 (0.9)	
Receiving benefits**				
Yes	2 (20.0)	3 (3.0)	5 (4.5)	0.064
No	8 (80.0)	97 (97.0)	105 (95.5)	
Employment status**				
Employed	3 (30.0)	76 (76.0)	79 (71.8)	
Unemployed	7 (70.0)	23 (23.0)	30 (27.3)	0.004
Missing	-	1 (1.0)	1 (0.9)	
Education, mother***				
Pre-secondary, ≤9 years	5 (50.0)	27 (27.0)	32 (29.1)	
Secondary, 10–12 years	4 (40.0)	34 (34.0)	38 (34.5)	0.739
Post-secondary,> 12 years	1 (10.0)	12 (12.0)	14 (12.7)	
Missing	-	30 (26.0)	26 (23.6)	
Education, father***				
Pre-secondary, ≤9 years	1 (10.0)	20 (20.0)	21 (19.1)	
Secondary, 10–12 years	3 (30.0)	37 (37.0)	40 (36.4)	1
Post-secondary, > 12 years	1 (10.0)	10 (10.0)	11 (10.0)	
Missing	5 (50.0)	33 (33.0)	38 (34.5)	

* Age in index year.

** Figure valid one year before the crime occurred.

*** Highest registered level of education.

Due to the small female perpetrator sample size, the decision was made not to proceed with bivariable analyses, as this might have generated a risk of false conclusions.

### Socio-demographic factors: Female perpetrators compared to female controls

Comparison of educational level and employment status revealed statistically significant differences between female perpetrators and female controls (Table 4). The majority of the female perpetrators (n = 7; 70%) were registered as unemployed, while the corresponding figure for the female controls was 23%. When it came to educational level in the female perpetrator group, none had a post-secondary education, whereas 38% of the female controls did.

## Discussion

### Male and female IPH perpetrators

This study identified 48 male and 10 female perpetrators during 2000–2016, a 17-year period. During the period one male perpetrator committed two IPH, on different occasions. These figures correspond well with national findings in Sweden [2]. The gender asymmetry among perpetrators was striking, as also reported in the literature [3,12,14,20,21]. The motive factors for committing IPH, as stated in the court records, were jealousy and threat of divorce/separation, which was reported in two-thirds of the male-perpetrated cases and in half of the female-perpetrated cases. This is in line with findings from other studies, i.e., that both male and female perpetration are linked to these factors [3,5,10,22]. The separation factor is, however, ambiguous. When it comes to the female-perpetrated cases in this study, two out of three women desired a separation, whereas their male partners did not. In the male-perpetrated cases, the female partners had wished to separate (some had started seeing a new partner).

Looking specifically at male and female perpetration in relation to IPV, this study found that IPV committed by the male partner was recurrent in the relationship and reported as a motivational factor in five out of ten female-perpetrated cases. In one of these cases, the woman was actually acquitted as she was judged to have acted in self-defence. Many studies confirm that male-to-female IPV is a risk factor for subsequent male-perpetrated IPH [5,10]. That being subjected to IPV occasionally results in female-perpetrated IPH, as a response to the violence inflicted, is discussed in the literature as a form of violent resistance [23–25].

A Danish retrospective study reported that strangulation and stabbing with a knife or other sharp object were the most common modes of killing, although blunt force also occurred [26]. Leth et al. found that the victims were strangled in almost one-third of all male-to-female IPH. There are some gendered differences; while both female and male perpetrators use sharp force, female perpetrators seldom strangle their victims [26,27]. This was also the case in our study, with the exception of one female perpetrator. Another study confirmed that knives were the most common weapon, but access to a gun was also a risk factor for male-perpetrated IPH [28].

The IPHS case rate in this study from the Västra Götaland Region of Sweden is slightly lower (16.3%) than in a previous study, in which the corresponding average figure was found to be 24% in Sweden during a nine-year period (1990–1999) [3]. However, it is unclear whether we have succeeded in identifying all the IPHS cases during the 17-year observation period for the purposes of this study. In the Swedish study by Belfrage and Rying, a comparison was made with perpetrators of all other types of homicide, and a 6% risk of suicide was found in this group, compared to 24% among IPH perpetrators [3]. Previous research shows that IPHS is mainly committed by male perpetrators [3,12,20,29]. Indeed, no female-perpetrated cases were found in this study. In Belfgrage and Rying’s study, forensic psychiatric assessments were performed in the majority of IPH cases and a high degree of psychiatric morbidity was identified [3], especially in IPHS cases. This concurs with other studies.

#### Male perpetrators

We found that one year before the crime occurred, male IPH perpetrators had lower disposable income and were to a higher extent unemployed and recipients of social benefits than the control group.

Caman revealed how IPH perpetrators are employed to a higher extent today than previously (4), as well as to a higher extent than non-partner-homicide perpetrators [30]. However, in comparison with the control group drawn from the general population, the perpetrators were unemployed more than twice as often at the time-point one year prior to the crime.

In the Västra Götaland Region, only 3.6% of the inhabitants (males and females) were benefits recipients [31] in 2016. However, this figure was about four times higher (20.8%), among the male perpetrators than among the male controls (4.8%). The circumstances leading to requiring benefits are most often unemployment, long-term illness and debts, as well as low educational level and low income, leading to the risk of falling below the poverty line. A US study investigated “county disadvantage”, which included median income, proportion of the inhabitants living below the poverty line, being unemployed, percentage of female-headed households, households with benefits recipients and those with less than high school education. That study found a statistically significant relationship between county disadvantage and male-perpetrated, female-victim IPH; one standard deviation increase in the county disadvantage score was associated with a 12% higher IPH rate (32).

We conclude that, although male IPH perpetrators are more conventional than non-intimate-partner homicide perpetrators as reported in previous research (14), this study found that they had a lower socio-demographic position in society, compared to the general population.

Higher unemployment rate, lower disposable income and receiving benefits are interlinked factors and they indicate a dependency in daily life, that may lead to stress and feelings of inferiority and frustration, which may in turn give rise to IPV and, in exceptional cases, to IPH. This is in line with the thinking behind the concept of county disadvantage, i.e., belonging to a disadvantaged group in the local society [32].

#### Female perpetrators

The vast majority (70%) of female perpetrators in this study were unemployed one year prior to the crime, compared to 23% in the female control group. This finding concurs with previous studies from Finland, Sweden and Norway, revealing how female perpetrators are commonly unemployed at the time of the crime [14,20,33]. Furthermore, the proportion of female perpetrators in paid employment has decreased over time in Sweden [4]. This could partly be explained by the fact that female IPH perpetrators may have been systematically subjected to IPV prior to the crime [4,9]. The individual effects of IPV are substantial, with difficulties working and isolation as two serious consequences that might contribute to explaining this higher unemployment rate. However, female perpetrators may also suffer from chronic physical or mental conditions or have alcohol- or drug-related problems, which may explain lower socio-demographic status and a lower degree of employment, as found in a study from Australia [34].

Finally, female perpetrators were less educated than female controls. Research findings on educational level among female IPH perpetrators are scarce. Leonard reported how convicted IPH female perpetrators had significantly higher educational levels than the average female prisoner [35]. Dugan and colleagues have theorised that higher education has protective effects against committing IPH, since it increases the opportunity for females to be employed and independent and thus terminate a difficult relationship [36]. The results of this study, in which female perpetrators were shown to have a lower level of education and were to a higher extent unemployed than the control group, are difficult to interpret with accuracy due to the small sample size but do suggest a population of vulnerable women.

### Methodological considerations

One limitation of this study is the small sample size, especially in the case of female perpetrators, making it difficult to detect differences and consequently increasing the possibility of a type-II errors. This in turn makes it difficult to generalise the findings to a larger population [37]. However, we decided to include the female sample descriptively, as research-based knowledge about female perpetrators is scarce. The male sample is also limited in size but did allow bivariable analyses.

There was a considerable amount of missing data for a few of the included variables, which was a limitation. However, the error rate was equal in both the control and perpetrator groups and there was no indication of a systematic difference between the groups.

The data presented in Table 1 were obtained from the court records and must be interpreted cautiously, especially regarding motivational factors, as they rely on the perpetrator’s account and on what the courts had found important to record. The court records differ in length and degree of detail, and the content depends on the topics that were the objects of inquiry. However, these documents provided information about the actual crime that is not available in any register.

The descriptive and bivariable analysis of the socio-demographic factors is based on a one-time measurement (index-1, index-3), and does not provide knowledge of any cause-effect relationship. The conclusion that IPH is caused by lower socio-demographic status can thus not be drawn. While this finding does add to existing knowledge of the importance of investigating socio-demographic factors, it is important to bear in mind that crimes such as lethal violence depend on many individual, relationship and structural level factors.

This is the first study to compare IPH perpetrators with a general population sample, which is an important strength.

Another strength was the inclusion of IPHS cases. Normally, when researchers examine cases of IPH, they include all cases with a court verdict and exclude all other potential IPH cases, especially the IPHS cases as they often lack a court verdict [20]. However, data on each IPHS case were obtained from the preliminary inquiries. This detailed research was made possible by good collaboration with the regional police authority.

Moreover, the use of register data eliminates the risk of the bias inherent in self-reporting. The register data in this study was attained from SCB, that has a quality certification [38].

## Conclusion

This study supports previous research findings when it comes to some of the investigated socio-demographic variables. However, it was performed with a control group representing a random sample of the general population rather than one selected for a history of abusive or violent behaviour. To the best of our knowledge, such a comparison has not been made before.

This study concludes that IPH perpetrators often are in a socio-economically disadvantaged position in society, including in comparison with a general population sample. In addition, this study shows a clear gender asymmetry in IPH, with male perpetration being the most frequent, in line with previous findings in Swedish and international research. The court records indicated that the male perpetrator is more likely to kill his female partner when she decides to leave him; at this moment her life is in real danger.

These findings can be used to enhance and develop preventive strategies. IPH perpetrators are often in contact with the criminal justice system, healthcare services or social and other support services during the year before the crime [10]. The fact that IPH is often preceded by IPV highlights the importance of healthcare and social services staff identifying cases of IPV perpetration and being aware of the risk of escalation. Healthcare and social services staff should ask about violence perpetration and be ready to act if IPV is revealed and they should be trained to assess the risk of IPH. Supportive intervention requires comprehensive assessments that not only include detection of IPV, but also focus on unemployment and economic situation, in order to identify relationships in which psychosocial life circumstances are poor and the risk of IPH perpetration is increased.
